# Covalently Functionalized DNA Duplexes and Quadruplexes as Hybrid Catalysts in an Enantioselective Friedel–Crafts Reaction

**DOI:** 10.3390/molecules25143121

**Published:** 2020-07-08

**Authors:** Surjendu Dey, Andres Jäschke

**Affiliations:** Institute of Pharmacy and Molecular Biotechnology, Heidelberg University, 69120 Heidelberg, Germany; surjendudey@gmail.com

**Keywords:** asymmetric catalysis, Friedel–Crafts reaction, hybrid catalysis, covalent modification, DNA

## Abstract

The precise site-specific positioning of metal–ligand complexes on various DNA structures through covalent linkages has gained importance in the development of hybrid catalysts for aqueous-phase homogeneous catalysis. Covalently modified double-stranded and G-quadruplex DNA-based hybrid catalysts have been investigated separately. To understand the role of different DNA secondary structures in enantioselective Friedel–Crafts alkylation, a well-known G-quadruplex-forming sequence was covalently modified at different positions. The catalytic performance of this modified DNA strand was studied in the presence and absence of a complementary DNA sequence, resulting in the formation of two different secondary structures, namely duplex and G-quadruplex. Indeed, the secondary structures had a tremendous effect on both the yield and stereoselectivity of the catalyzed reaction. In addition, the position of the modification, the topology of the DNA, the nature of the ligand, and the length of the linker between ligand and DNA were found to modulate the catalytic performance of the hybrid catalysts. Using the optimal linker length, the quadruplexes formed the (−)-enantiomer with up to 65% *ee*, while the duplex yielded the (+)-enantiomer with up to 62% *ee*. This study unveils a new and simple way to control the stereochemical outcome of a Friedel–Crafts reaction.

## 1. Introduction

The Friedel–Crafts reaction was first reported in 1877 [1,2]. Since then, it has become one of the most versatile methods for the formation of carbon–carbon bonds, and enantioselective Friedel–Crafts alkylation reactions have gained particular importance [3,4,5]. While the classical Friedel–Crafts reactions were performed under strictly anhydrous conditions, in recent years numerous attempts were made to expand this reaction to aqueous media [6]. Among other strategies, hybrid catalysis is an emerging concept in aqueous-phase homogeneous asymmetric catalysis [7,8,9,10]. Here, catalytically active transition metal complexes are embedded in the chiral scaffold of biopolymers, such as proteins or nucleic acids, which behave as a second coordination sphere for increased reactivity and/or enantioselectivity [11,12,13,14,15,16]. Thus, the structure of biopolymers plays a crucial role in catalysis.

The concept of DNA-based hybrid catalysts was first reported by Roelfes and Feringa in 2005, where the combination of a double-stranded (ds) DNA and a Cu(II)-complex covalently bound to an intercalating moiety was tested [17]. Since then, this approach has been successfully expanded to various enantioselective reactions, such as Diels–Alder reactions [17,18,19,20,21], inverse electron-demand hetero-Diels–Alder reactions [22], Friedel–Crafts alkylations [23,24,25,26,27], Michael additions [28,29,30], fluorinations [31], *syn*-hydrations [32], and metal–organic reactions [33]. In all cases, a non-covalent strategy was used to combine the dsDNA and different Cu(II)–ligand complexes. Besides dsDNA, other nucleic acid scaffolds, such as DNA hairpins [34], hybrid DNA/RNA or dsRNA [35], and peptide nucleic acid (PNA) [36], were also used to construct enantioselective catalysts. Recently, a cyclic dinucleotide-based catalyst [37] and a guanosine-based self-assembly [38] were reported for enantioselective Friedel–Crafts reactions.

Among the various nucleic acid scaffolds, the G-quadruplex family is quite promising owing to its high structural diversity, which makes it a favorable candidate for the development of hybrid catalysts [39,40,41]. Two well-known G-quadruplex-forming sequences, namely the human telomeric G-quadruplex (h-Tel), and a region of the c-kit promoter (c-kit) in combination with Cu(II) ions, have been successfully applied to asymmetric Diels–Alder, Friedel–Crafts and sulfoxidation reactions [42,43,44,45,46,47,48]. Among these two quadruplexes, h-Tel showed higher catalytic activity and stereoselectivity. In addition, an assembly of G-triplex DNA and Cu(II) was utilized in an asymmetric Diels–Alder reaction, but showed only moderate enantioselectivity [49]. In most of these studies, the binding of Cu(II) to the G-quadruplex sequences was assumed to be unspecific and probably due to electrostatic interactions. The nature of these interactions makes precise positioning of the catalytic metal ion relative to the bound substrate impossible, which in turn prevents the prediction of the parameters that determine activity and selectivity in these reactions. To overcome these problems, a site-specific covalent attachment strategy can be an alternative, allowing the covalent anchoring of the transition-metal ligand complex to the DNA in a precise manner, thus enabling the understanding of structure–function relationships [50,51,52,53,54,55,56,57,58,59,60].

Previously, we reported a new type of efficient and stereoselective hybrid catalyst and its application to asymmetric Michael additions in water [61,62]. In these catalysts, c-kit G-quadruplex DNA was site-specifically linked—via covalent tethers—to bipyridyl (bpy) ligands coordinated to Cu(II) ions. We observed that the stereoselectivity of the reaction can be easily tuned by choosing different attachment positions on the DNA sequence. We furthermore found that several different factors, such as the position of modification, the topology of the quadruplex, the nature of the ligand, and the length of the linker between the ligand and DNA, dictate the catalytic activity and stereoselectivity.

Herein, we report a direct comparison of G-quadruplex and duplex DNA-based hybrid catalysts, formed by covalent attachment of different bpy-linker constructs to the quadruplex forming sequences and their application on an enantioselective Friedel–Crafts alkylation. Surprisingly, the stereoselectivity could be tuned by selecting an appropriate linker length and DNA structure. While quadruplexes preferentially formed the (−)-enantiomer with up to 65% *ee*, the best duplex yielded the (+)-enantiomer with up to 62% *ee*.

## 2. Results and Discussion

### 2.1. Synthesis

**Figure 1:**
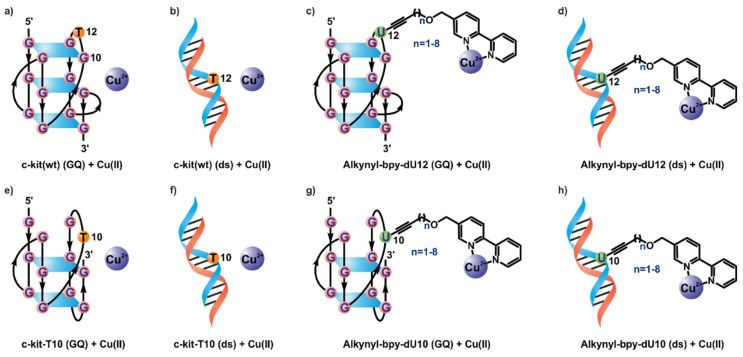
Folding of different G-quadruplex and dsDNA sequences in the presence of Cu(II). (**a**) Folding of (GQ) c-kit(wt) DNA as observed [63,64]. (**b**–**h**) Schematic representation of hypothetical folding and metal binding of (ds) c-kit(wt) (**b**), (GQ) dU12-modified DNA (**c**), (ds) dU12-modified DNA (**d**), (GQ) c-kit-T10 DNA (**e**), (ds) c-kit-T10 DNA (**f**), (GQ) dU10-modified DNA (**g**), and (ds) dU10-modified DNA (**h**). All double-stranded (ds) samples included 1 equivalent corresponding complementary DNA strand.

For our investigation, we chose the c-kit wild-type (wt) sequence (5′-AGGGAGGGCGCT-GGGAGGAGGG-3′), which forms a unique all-parallel G-quadruplex structure according to high-resolution structural data from X-ray crystallography [63] and NMR spectroscopy [64] (as shown in Figure 1a). Based on this structural information and our previous experience [61,62], positions 12 and 10 were picked as sites of attachment for the bpy ligand. As position 12 was located in an apical loop segment, the influence of a bulky substitution was not noticeable according to crystallographic studies [63]. On the other hand, position 10 was highly important to the formation of the unique all-parallel folding topology. Mutating G10 with T, namely c-kit-T10, favored an alternative quadruplex folding, as observed in CD-spectroscopic measurements [64]. While the exact structure of this quadruplex is unknown, our previous studies suggest a mixed parallel–antiparallel topology [61,62,64]. A hypothetical structure of the c-kit-T10 mutant is drawn in Figure 1e. Both quadruplex-forming sequences can be turned into double-stranded structures by the addition of a full-length complementary DNA strand. We systematically substituted the thymidine residues T12 and T10 by various deoxyuridine-bpy conjugates differing only in the number of sp^3^-hybrized carbon atoms within the linker (between 0 and 8). The respective phosphoramidites were synthesized as described earlier [61,62]. The modified DNA strands were synthesized by solid-phase deoxyoligonucleotide synthesis and purified by HPLC (Figure S1, see the Supporting Information for details).

### 2.2. Catalytic Properties

To investigate the catalytic activity of the modified G-quadruplexes (GQ) and dsDNA with covalently linked bpy-moieties in combination with Cu(II), a model enantioselective Friedel–Crafts reaction of (*E*)-1-(1-methyl-1*H*-imidazol-2-yl)but-2-en-1-one (**1**) and 5-methoxy-1*H*-indole (**2**) was chosen [23]. The catalysts were prepared in situ by the combination of 2.5 mol% Cu(NO_3_)_2_ (25 μM) and a slight excess of bpy-linked DNA (33.3 μM).

First, we examined the reaction utilizing different dU12-modified G-quadruplexes in combination with Cu(II) (Table 1, entries 3–10). While propargyl-bpy and butynyl-bpy showed relatively lower conversion (Table 1, entries 3 and 4), longer linkers (n > 2) yielded almost quantitative conversion (Table 1, entries 5–10). In most of the cases, the (−)-enantiomer was favoured with up to −65% *ee* using a pentynyl-bpy linker (Table 1, entry 5). However, reversal of stereoselectivity was observed for the octynyl and decynyl linkers with +29% and +8% *ee,* respectively (Table 1, entries 8 and 10). Furthermore, a series of control experiments were performed utilizing either Cu(NO_3_)_2_ (Table 1, entries 1 and 2, and Table S1) or different Cu(II)-complexes (Table S2) in the absence or presence of c-kit(wt) or c-kit-T10 (both the GQ and dsDNA). In the case of Cu(NO_3_)_2_, low conversion with almost negligible stereoinduction (Table 1, entries 1 and 2; Table S1) was noticed. On the other hand, low to moderate conversion along with very low stereoselectivity (always in favour of the (+)-enantiomer) was obtained by employing different Cu(II)-complexes (Table S2). These results confirm the importance of the covalent bpy-linker attachment to the DNA, which enhances the catalytic efficiency and also shows that the stereoinduction is instigated by the topology of the DNA scaffold, and not by the chirality of the modified nucleoside.

Intrigued by these results, we changed the DNA structure from G-quadruplex to double-stranded. For these experiments, the dU12-modified sequences were combined with a complementary DNA sequence to fold into the dsDNA, which was used as a catalyst in combination with Cu(II) for the same enantioselective Friedel–Crafts alkylation. Again, an increase in conversion starting from 27% for propargyl-bpy (Table 1, entry 3) to almost quantitative for heptynyl-bpy and longer linkers (Table 1, entries 7–10) was achieved. Surprisingly, the stereoselectivity obtained by applying linker lengths n ≤ 4 and n = 8 for the dU12-modified dsDNA was opposite to that obtained with the dU12-modified quadruplex for the same linkers (Table 1, entries 3–6 and 10). In contrast, for the linker lengths (n = 5–7), both the dU12-modified quadruplex and dsDNA showed similar stereoselectivity for the product formation (Table 1, entries 7–9).

Next, we tested the dU10-modified quadruplexes which are likely to fold differently based on our previous CD measurements [61,62] (Figures S2 and S3). These quadruplexes gave significantly lower yields than the dU12-GQs, ranging from 44% to 68% and never approaching quantitative yields (Table 1, entries 3–10). In all cases, the (+)-enantiomer was obtained in excess with up to +45% *ee* (Table 1, entry 4). Remarkably, the preferentially formed enantiomer was always opposite to that obtained with the dU12-modified quadruplexes, except for the octynyl-bpy and the decynyl-bpy linkers (Table 1, entries 8 and 10).

The dU10-modified dsDNA was our next focus. In this case, a steady increase in conversion was observed with increasing linker length (Table 1, entries 3–10). Again, the (+)-enantiomer was obtained in excess in all cases. Here, the nonynyl-bpy linker was found to induce the highest stereoselectivity (+53% *ee*).

**Figure 2:**
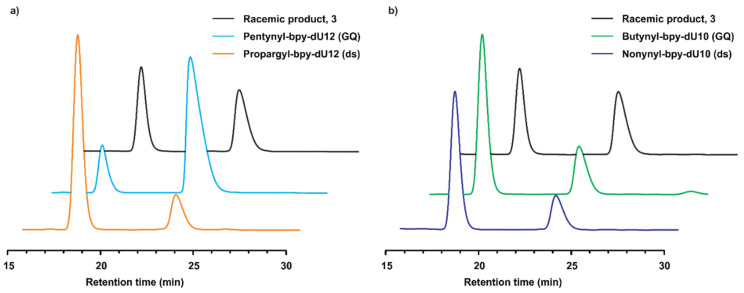
Comparison of chiral HPLC chromatograms of product 3. (**a**) Comparison of the Friedel–Crafts alkylation product 3 using different dU12-modified DNA structures. (**b**) Comparison of the Friedel–Crafts alkylation product 3 using different dU10-modified DNA structures.

From the systematic studies of different dU12 and dU10-modified quadruplexes and dsDNA in the enantioselective Friedel–Crafts product formation, the roles of the bpy-linker modification and the DNA secondary structure are clearly visible. The optimal bpy-linker for achieving high enantioselectivity depends on the DNA structure. In the case of the dU12 modification, the pentynyl-bpy (−65% *ee*) and the propargyl-bpy (+62% *ee*) linkers were found to be more efficient for the quadruplex and dsDNA, respectively (Figure 2a). However, the butynyl-bpy (+45% *ee*) and the nonynyl-bpy (+53% *ee*) linkers more strongly induced stereoselectivity in the case of the dU10 modification with quadruplex and dsDNA structures, respectively (Figure 2b). Comparing all results, three trends were revealed with respect to the percentage of product formation. In the case of small linkers (n ≤ 3), the trend GQ(dU12)> GQ(dU10)> ds(dU12)> ds(dU10) was noticed (Table 1, entries 3–5). Using the hexynyl linker (n = 4), the order became GQ(dU12)> ds(dU12)> GQ(dU10)> ds(dU10) (Table 1, entry 6). Surprisingly, a third trend GQ(dU12)> ds(dU12)> ds(dU10)> GQ(dU10) was also observed for n ≥ 4 (Table 1, entries 7–10).

In the above experiments, the bpy ligand was connected to the deoxyuridine using different flexible linkers with up to ten rotatable atoms (sp^3^-hybridized C, ether-O), which contribute large translational and rotational degrees of freedom. Thus, the options for positioning the ligand with respect to the DNA were limited only by the length of the linker. Therefore, we extended our study by introducing a rigid linker in which an ethynyl bridge between the bpy ligand and C5-position of the deoxyuridine was chosen (Figure S4) [62,65,66,67,68]. An ethynyl bridge offers no translational and very little rotational degrees of freedom compared to the flexible systems. By applying the ethynyl-bpy modified dU12 and dU10 quadruplexes in the Friedel–Crafts alkylation, the (+) enantiomer was observed in excess and showed +45% and +30% *ee*, respectively (Table 1, entry 11). However, the corresponding dsDNA yielded lower enantioselectivity with the (−) enantiomer in excess. In this case, tuning of stereoselectivity is clearly due to the change in DNA structure. Comparing the results of the percentage of product formation (Table 1, entry 11), the trend GQ(dU12)> GQ(dU10)>ds(dU12)>ds(dU10) was observed, which is similar to the small flexible linkers (n ≤ 3) (Table 1, entries 3-5). Remarkably, the conversion yield utilizing the rigid ethynyl bridge is similar to the more flexible propargyl derivative, however the enantioselectivity results are different (compare entries 3 and 11 in Table 1). These trends suggest that the respective catalytic centers have different architectures. However, in the absence of high-resolution structural data and absolute stereochemistries, it is impossible to pinpoint the molecular reasons for these differences.

Looking at rate acceleration and stereoselectivity at the same time, the propargyl-bpy and the ethynyl-bpy derivatives (except for the dU12-GQ) can hardly be called “catalysts”, as there is no rate acceleration, compared to the unmodified DNA strands (compare lanes 3 and 11 with 1 and 2 in Table 1). It is questionable whether in these cases the catalytic copper ion is at all coordinated to the bpy ligand. The moderate to high stereoinduction in these cases may be solely due to restricted access to the catalytic center which could be a copper ion coordinated directly to the DNA. Starting with butynyl, the reaction rate increases significantly in all four formats, indicating that at least two sp^3^-hybridized carbon atoms in the linker are required for the formation of an efficient hybrid catalyst that accelerates the reaction and modulates the stereochemistry. In these cases, the catalytic center is likely composed of copper, the bpy ligand, and heteroatoms of specific nucleobases.

While the different enantioselectivities of the two different quadruplex topologies (dU12 vs. dU10) are in agreement with our previous work on DNA-catalyzed Michael additions [61,62], the large difference in reaction yields was unexpected, indicating that the relations uncovered here cannot be easily generalized and extended to other chemical transformations. Furthermore, the different reaction yields and partly opposite enantioselectivities of the two very similar DNA duplexes (dU12 vs. dU10) are surprising. The major difference between these two duplexes is that the bpy-linker-modified dU residue is located between two C residues in the case of dU10, while it is between a C and a G in dU12. The latter system consistenly delivers higher reaction yields and mostly higher levels of stereoselectivity. Thus, both for DNA quadruplexes and duplexes the direct vicinity of the catalytic center has a large influence on the catalytic performance.

## 3. Conclusions

In summary, we found that DNA sequences covalently modified with linker-bpy-Cu(II) complexes can be efficient catalysts for enantioselective Friedel–Crafts alkylation. Our experiments suggest that the secondary structures of DNA, the position of modification, the topology of the DNA, the nature of the ligand, and the length of the linker between the ligand and the DNA are all important factors for the catalytic efficiency. Applying these hybrid catalysts, stereochemical outcomes of the reaction can be tuned. The quadruplex DNA yielded the (−)-enantiomer with up to 65% *ee*, however double-stranded DNA provided the (+)-enantiomer with up to 62% *ee*. Furthermore, our experiments show that the dependence of linker length on catalyst performance is far from trivial and even very long, flexible linkers can still lead to potent and selective catalysts. Finally, we believe that our findings open a new avenue to construct efficient DNA-based hybrid catalysts, which may develop into useful tools for asymmetric catalysis under environmentally benign conditions.

## Figures and Tables

**Table 1:**
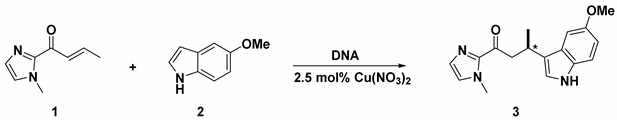
Results of the Friedel–Crafts alkylation reaction catalyzed by dU12/dU10-modified c-kit DNA and Cu(II).^a^

Entry	DNA/Linker	n	dU12-modified DNA				dU10-modified DNA			
			GQ-DNA		ds-DNA		GQ-DNA		ds-DNA	
			conv (%) ^b^	*ee* (%) ^b, c^	conv (%) ^b^	*ee* (%) ^b, c^	conv (%) ^b^	*ee* (%) ^b, c^	conv (%) ^b^	*ee* (%) ^b, c^
1	c-kit(wt)	-	34	<−5	28	<+5	-	-	-	-
2	c-kit-T10	-	-	-	-	-	45	<+5	21	<+5
3	Propargyl-bpy	1	64	−23	27	+62	44	+17	23	+37
4	Butynyl-bpy	2	78	−35	48	+16	64	+45	36	+21
5	Pentynyl-bpy	3	99	−65	63	+25	68	+14	49	<+5
6	Hexynyl-bpy	4	99	−19	79	+8	65	+16	61	+31
7	Heptynyl-bpy	5	99	−22	99	−8	67	+10	88	+33
8	Octynyl-bpy	6	99	+29	99	+7	66	+21	89	+40
9	Nonynyl-bpy	7	99	−13	99	−23	64	+8	89	+53
10	Decynyl-bpy	8	99	+8	99	−11	65	+7	91	+38
11	Ethynyl-bpy	-	68	+45	28	<−5	45	+30	21	−19

^a^ See the Experimental Section for detailed reaction condition. All experiments were performed in triplicate. ^b^ Both conversion and *ee* were calculated by using chiral HPLC; results are reproducible within ±5%. ^c^ (+) and (−) symbols refer to isomers with low and high retention times, respectively, from the chiral HPLC column.

## Supplementary Materials

The following are available online at https://www.mdpi.com/1420-3049/25/14/3121/s1, Figure S1: HPLC chromatograms of modified oligonucleotides [2,3], Figure S2: CD-spectra of c-kit(wt) oligonucleotide modified at position 12 in 20 mM MOPS (pH=7) + 100 mM KCl. All formed G-quadruplexes [2,3], Figure S3: CD-spectra of c-kit oligonucleotide modified at position 10 in 20 mM MOPS (pH=7) + 100 mM KCl. All formed G-quadruplexes [2,3], Figure S4. Folding of different G-quadruplex and dsDNA sequences in the presence of Cu(II), Table S1: Friedel-Crafts alkylation reaction using different catalytic conditions [a], Table S2:Friedel-Crafts alkylation reaction using different catalytic conditions [a].
