# Supplementary material for: Covalently Functionalized DNA Duplexes and Quadruplexes as Hybrid Catalysts in an Enantioselective Friedel–Crafts Reaction
Source: Molecules. 2020 Jul 8;25(14):3121. doi: 10.3390/molecules25143121 (PMC7397069; doi:10.3390/molecules25143121)
Supplement: Supplementary file 1 [file molecules-25-03121-s001.pdf]

Supporting information

Covalently functionalized DNA duplexes and quadruplexes as hybrid catalysts in an enantioselective Friedel-Crafts reaction

Surjendu Dey and Andres Jäschke*

Institute of Pharmacy and Molecular Biotechnology, Heidelberg University, 69120 Heidelberg, Germany.

Correspondence should be addressed to AJ. (jaeschke@uni-hd.de)

Contents

1 General materials and methods 1

2 Synthesis of nucleoside and phosphoramidite derivatives 3

3 Used DNA sequences 3

4 Synthesis and purification of DNA sequences 4

4.1 Analysis of synthesized DNA sequences 4

4.2 LC-MS analysis results of the synthesized DNA 5

4.3 HPLC chromatograms 6

5 Standard Friedel-Crafts reaction procedure 8

5.1 Reaction using G-quadruplex DNA 8

5.2 Reaction using double-stranded DNA 8

5.3 Separation of product enantiomers by chiral HPLC 9

5.4 Reaction control-1 9

5.5 Reaction control-2 10

6 CD spectroscopy 12

7 Ethynyl-bpy modified DNA 17

8 References 18

1 General materials and methods

5-Iodo-2'-deoxyuridine was purchased from Carbosynth. All other chemicals were purchased from Sigma-Aldrich or ABCR and used without further purification. Non-modified oligonucleotides were purchased from Biomers.net GmbH and Integrated DNA Technologies. For the purification of synthesized oligonucleotides, semi-preparative HPLC was carried out on an Agilent 1100 Series HPLC system equipped with a diode array detector (DAD) using a Phenomenex Luna C18 column (5 µm, 250 X 15.0 mm) at a flow rate of 5 mL/min using a gradient of buffer A (100 mM triethylammonium acetate pH 7.0) and buffer B (100 mM triethylammonium acetate in 80% acetonitrile). Analytical HPLC was performed on the same HPLC system using a Agilent Poroshell 120 SB-C18 column (2.7 µm, 100 X 4.6 mm) with a flow rate of 1 mL/min and elution was performed with a gradient of buffer A and buffer B. LC-MS experiments were performed on a Bruker microTOF-Q II ESI mass spectrometer connected to an Agilent 1200 Series HPLC system equipped with a multi-wavelength detector (MWD). A Phenomenex kinetex C18 column (2.6 µm, 100 X 2.1 mm) was used with a flow rate of 0.2 mL/min and eluting with a gradient of buffer C (100 mM hexafluoroisopropanol + 8.6 mM trimethylamine, pH 8.3) and methanol (LC-MS grade). Analysis of the LC-MS measurements was performed using Hyphenation Star PP (Version 3.2.44.0) and Data Analysis (Version 4.0, SP 4) software (Bruker Daltonics). Recorded MS-spectra were deconvoluted using Maximum Entropy deconvolution. For high-resolution mass spectra, internal calibration was performed (enhanced quadratic mode) using Agilent Low Concentration Tuning Mix as a calibrant. Calculated molecular weights refer to the m/z values given by the Data Analysis software. Chiral HPLC was performed on an Agilent 1100 Series HPLC system equipped with a variable wavelength detector (VWD) using a Chiralpak AD-H column (5 µm, 250 X 4.6 mm) with a flow rate of 1 mL/min and eluting isocratically with n-hexane and isopropanol (LC-MS grade) 80:20. CD spectra were recorded on a Jasco (model J-810) spectropolarimeter. NMR spectra were recorded on Varian Mercury Plus 500 MHz spectrometer and Varian Mercury Plus 300 MHz spectrometer. Oligonucleotide synthesis was performed on an Expedite™8909 automated synthesizer using standard reagents from Sigma Aldrich Proligo.

Synthesis of (*E*)-1-(1-methyl-1*H*-imidazol-2-yl)but-2-en-1-one (1):

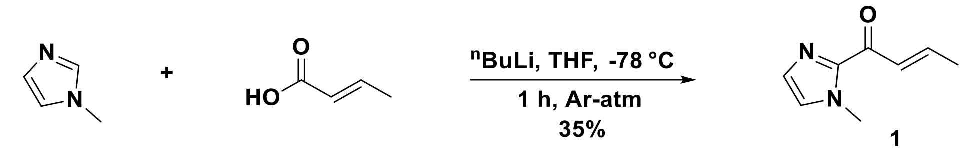

Synthesized according to the published procedure.[1]

In a 250 mL Schlenk flask, 160 ml dry THF and 1-methylimidazole (7.1 ml, 89 mmol, 2.2 eq.) were added and cooled down to -78 °C. Then, nBuLi 2.5 M in hexanes (35.6 mL, 89 mmol, 2.2 eq.) was dropwise added. The reaction was stirred at -78 °C for 5 min and then the cooling bath was removed, the reaction was allowed to return to RT over 30 min. The reaction was again cooled down to -78 °C. After that, a solution of *trans*-crotonic acid (3.5 g, 40 mmol, 1 eq.) in 20 ml dry THF was added dropwise. The reaction was stirred for 10 min at -78 °C, allowed to return to RT, and stirred for 30 min at RT. The reaction was quenched by dropwise addition of 60 ml saturated $NaHCO_3$ solution. The aqueous layer was extracted with EtOAc (3 x 100 mL) and dried over Na_2SO_4, then the solvent was evaporated. The crude product was purified by flash chromatography (40% EtOAc/hexane), which afforded yellowish oily product (2.1 g, 13.98 mmol, 35%).

^{1}H NMR (300 MHz, $CDCl_3$): δ 7.39 (dq, *J* = 15.5, 1.6 Hz, 1H), 7.17-7.05 (m, 2H), 7.02 (s, 1H), 4.02 (s, 3H), 1.96 (dd, *J* = 6.9, 1.6 Hz, 3H).

^{13}C NMR (75 MHz, $CDCl_3$): δ 180.5, 143.7, 129.0, 127.7, 126.9, 36.2, 18.3.

2 Synthesis of nucleoside and phosphoramidite derivatives

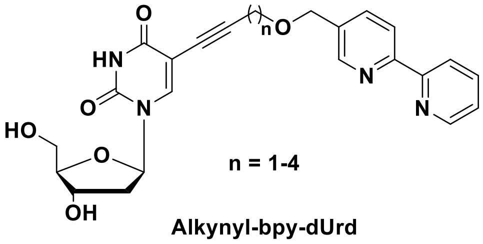

Alkynyl-bpy-dUrd

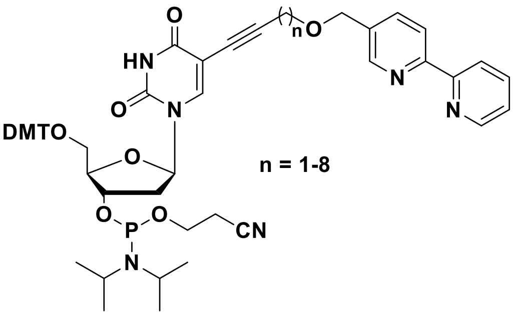

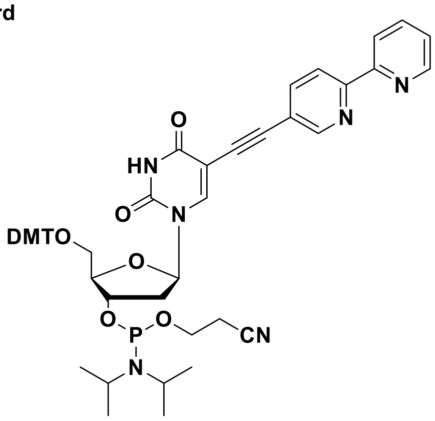

Alkynyl-bpy-DMTdU phosphoramidites **Ethynyl-bpy-DMTdU phosphoramidite**

All the alkynyl-bpy-dUrd derivatives, alkynyl-bpy-DMTdU phosphoramidites and ethynyl-bpy-DMTdU phosphoramidite were synthesized as described previously.[2-4]

3 Used DNA sequences

c-kit(wt): 5'-AGG GAG GGC GCT GGG AGG AGG G-3'
c-kit-T10: 5'-AGG GAG GGC TCT GGG AGG AGG G-3'

Linker-bpy attached dU12-DNA: 5'-AGG GAG GGC GCdU* GGG AGG AGG G-3'
Linker-bpy attached dU10-DNA: 5'-AGG GAG GGC dU*CT GGG AGG AGG G-3'
where, dU* is the linker-bpy attached deoxyuridine derivatives

cDNA-1 (comp. for c-kit(wt) & dU12-DNA sequences): 5'-CCC TCC TCC CAG CGC CCT CCC T-3'
cDNA-2 (comp. for c-kit-T10 & dU10-DNA sequences): 5'-CCC TCC TCC CAG AGC CCT CCC T-3'

4 Synthesis and purification of DNA sequences

Solid phase oligonucleotide synthesis was performed using the standard 1 µmol synthesis protocol for 5'-DMT-ON mode. Oligonucleotides were synthesized using standard reagents purchased from Sigma Aldrich Proligo on an Expedite™8909 automated synthesizer. All standard phosphoramidites and self-synthesized phosphoramidites (shown above) were diluted to 0.075 M and 0.1 M, respectively, for the synthesis. Oligonucleotides were deprotected and cleaved from the solid support using 27% aq. NH_3 solution and purified by semi-preparative HPLC (see general materials and methods). The collected product fractions were lyophilized, detrilytated using 2% aq. trifluoroacetic acid (TFA) solution, then neutralized with saturated aq. $NaHCO_3$ solution and then precipitated with ^{i}PrOH. The purity of the oligonucleotides was analyzed by both analytical HPLC and LC-MS.

Semi-preparative HPLC method

Time (min)	% buffer B	% buffer A	Flow (mL/min)
0	10	90	5
5	20	80	5
10	30	70	5
35	40	60	5
40	100	0	5
42	10	90	5

Analytical HPLC method

Time (min)	% buffer B	% buffer A	Flow (mL/min)
0	5	95	1
10	10	90	1
30	35	65	1
35	100	0	1
40	100	0	1
45	5	95	1
50	5	95	1

4.1 Analysis of synthesized DNA sequences

LC-MS analyses were performed on a Bruker microTOF-Q II ESI mass spectrometer connected to an Agilent 1200 Series HPLC system as described above. Optimized ESI source parameters were as follows: End Plate Offset -500 V, Capillary Voltage 4500 V, Nebulizer 1.2 Bar, Dry Gas 6.0 L/min, Dry Temperature 220 °C, Collision Energy 10.0 eV, negative ion mode.

Time (min)	% MeOH	% buffer C	Flow (mL/min)

0	5	95	0.2
30	35	65	0.2
35	100	0	0.2
40	5	95	0.2
50	5	95	0.2

4.2 LC-MS analysis results of the synthesized DNA

DNA	Molecular formula [M]	Retention time [min]	Calculated [M]	Deconvoluted [M]
Propargyl-bpy-dU10	$C_{231}H_{275}N_{102}O_{129}P_{21}$	20.6	7194.2680	7194.2538
Propargyl-bpy-dU12	$C_{231}H_{274}N_{105}O_{128}P_{21}$	21.0	7219.2745	7219.2532
Butynyl-bpy-dU10	$C_{232}H_{277}N_{102}O_{129}P_{21}$	21.2	7208.2837	7208.2689
Butynyl-bpy-dU12	$C_{232}H_{276}N_{105}O_{128}P_{21}$	21.1	7233.2901	7233.2733
Pentynyl-bpy-dU10	$C_{233}H_{279}N_{102}O_{129}P_{21}$	21.6	7222.2993	7222.2721
Pentynyl-bpy-dU12	$C_{233}H_{278}N_{105}O_{128}P_{21}$	21.0	7247.3058	7247.2867
Hexynyl-bpy-dU10	$C_{234}H_{281}N_{102}O_{129}P_{21}$	22.2	7236.3150	7236.2702
Hexynyl-bpy-dU12	$C_{234}H_{280}N_{105}O_{128}P_{21}$	21.4	7261.3215	7261.2817
Heptyny-bpyl-dU10	$C_{235}H_{283}N_{102}O_{129}P_{21}$	22.7	7250.3306	7250.3321
Heptyny-bpyl-dU12	$C_{235}H_{282}N_{105}O_{128}P_{21}$	22.6	7275.3371	7275.3147
Octynyl-bpy-dU10	$C_{236}H_{285}N_{102}O_{129}P_{21}$	23.2	7264.3463	7264.3206
Octynyl-bpy-dU12	$C_{236}H_{284}N_{105}O_{128}P_{21}$	23.1	7289.3528	7289.3213
Nonynyl-bpy-dU10	$C_{237}H_{287}N_{102}O_{129}P_{21}$	23.1	7278.3619	7278.3309
Nonynyl-bpy-dU12	$C_{237}H_{286}N_{105}O_{128}P_{21}$	24.2	7303.3684	7303.3406
Decynyl-bpy-dU10	$C_{238}H_{289}N_{102}O_{129}P_{21}$	25.4	7292.3776	7292.3433
Decynyl-bpy-dU12	$C_{238}H_{288}N_{105}O_{128}P_{21}$	24.7	7317.3841	7317.3432
Ethynyl-bpy-dU10	$C_{229}H_{271}N_{102}O_{128}P_{21}$	19.4	7150.2418	7150.2444
Ethynyl-bpy-dU12	$C_{229}H_{270}N_{105}O_{127}P_{21}$	19.6	7175.2483	7175.2521

4.3 HPLC chromatograms

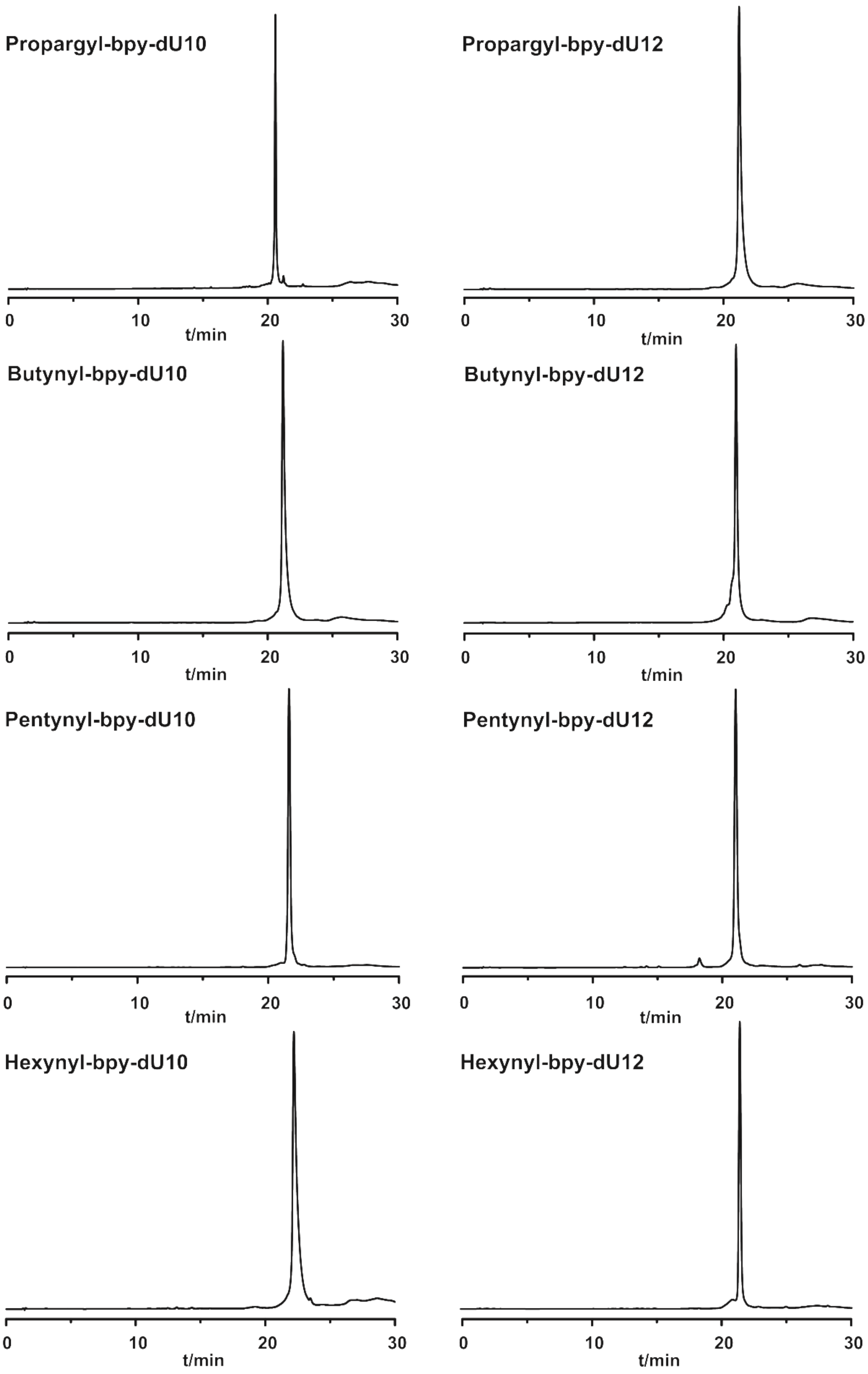

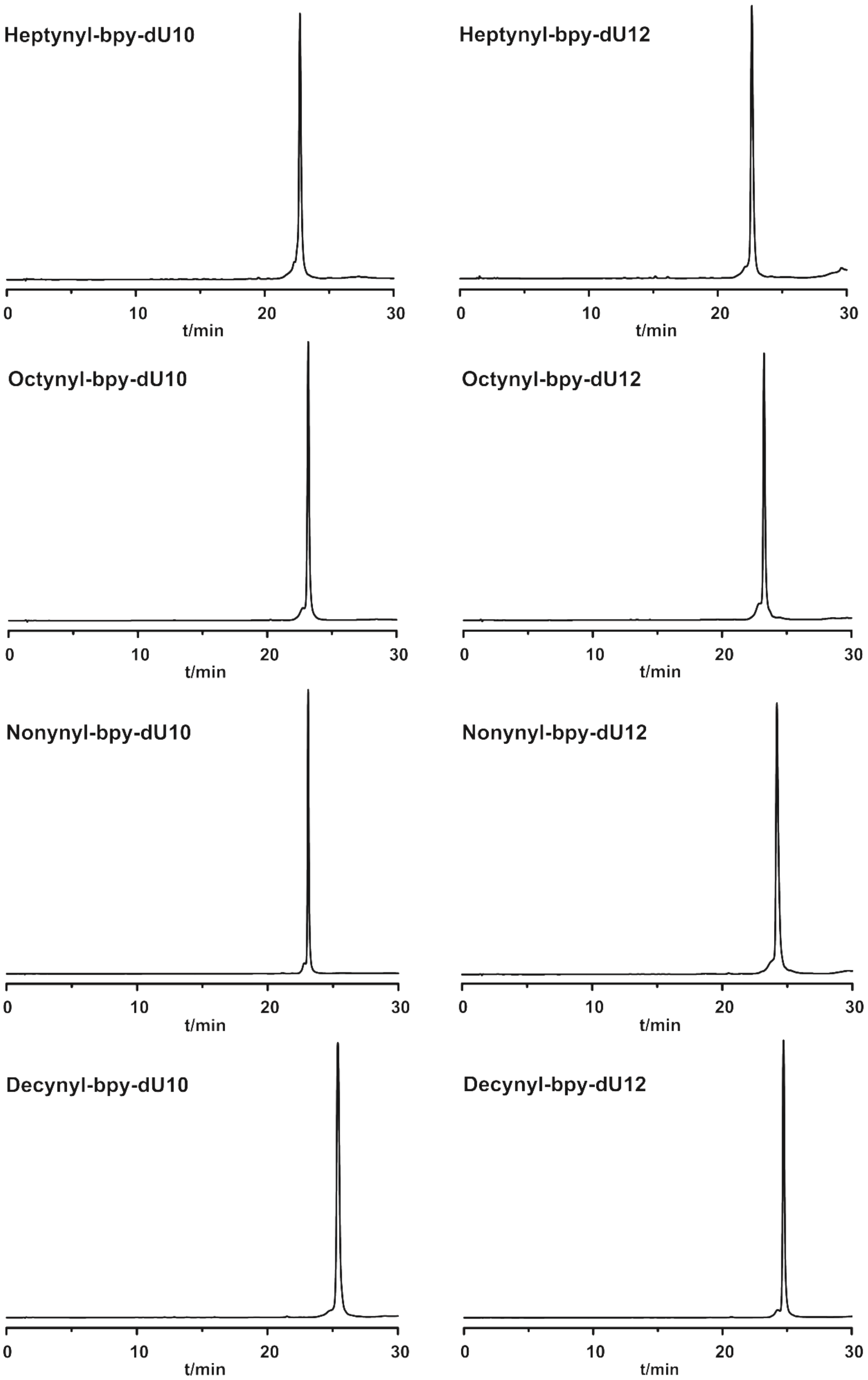
Heptynyl-bpy-dU10
Heptynyl-bpy-dU12
Octynyl-bpy-dU10
Octynyl-bpy-dU12
Nonynyl-bpy-dU10
Nonynyl-bpy-dU12
Decynyl-bpy-dU10
Decynyl-bpy-dU12
0
10
20
30
t/min

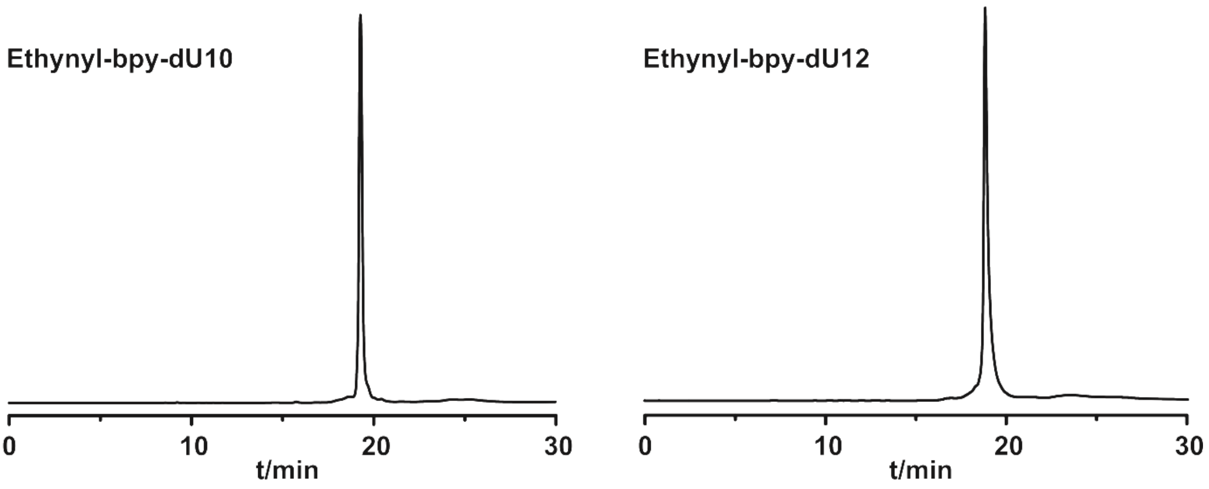

Figure S1: HPLC chromatograms of modified oligonucleotides.[2,3]

5 Standard Friedel-Crafts reaction procedure

5.1 Reaction using G-quadruplex DNA

An aqueous stock solution containing 10 nmol of the DNA was lyophilized first. Then the lyophilized DNA was dissolved in 297 µL of a solution of 3-(N-morpholino)propanesulfonic acid (MOPS) buffer (20 mM, pH 7) containing KCl (100 mM). 3 µl $Cu(NO_3)_2$ (2.5 mM) solution was added additionally. Final DNA and Cu(II) concentration was 33.3 µM and 25 µM, respectively. The solution was heated for 5 min at 90 °C, and slowly cooled down to room temperature. The solution was kept at 5 °C overnight before use. To that catalyst solution 3 µL of a fresh stock solution of (*E*)-1-(1-methyl-1*H*-imidazol-2-yl)but-2-en-1-one (**1**) (100 mM) in DMSO was added, followed by the addition of 3 µL (250 mM in DMSO, 2.5 eq.) 5-methoxy-1*H*-indole (**2**). The reaction mixture was stirred for 15 h at 5 °C. Extraction was performed with Et_2O (3 x 500 µL). After drying the organic phase with anhydrous Na_2SO_4, the solvent was removed to obtain the crude product. The crude product was directly injected to chiral HPLC to obtain the conversion and enantiomeric excess.

5.2 Reaction using double-stranded DNA

An aqueous stock solution containing 10 nmol of DNA and 10 nmol of corresponding complementary sequence (cDNA-1 or cDNA-2) were lyophilized first. Then the lyophilized dsDNA was dissolved in a 297 µL solution of 3-(N-morpholino)propanesulfonic acid (MOPS) buffer (20 mM, pH 7) containing KCl (100 mM). 3 µl $Cu(NO_3)_2$ (2.5 mM) solution was added additionally. Afterwards the reaction was performed as described above.

5.3 Separation of product enantiomers by chiral HPLC

Chiral HPLC was performed on an Agilent 1100 Series HPLC system equipped with a variable wavelength detector (VWD) using a Chiralpak AD-H column (5 µm, 250 X 4.6 mm) with a flow rate of 1 mL/min and eluting isocratically with n-hexane and isopropanol (LC-MS grade) 80:20. Detection wavelength was 254 nm.

5.4 Reaction control-1

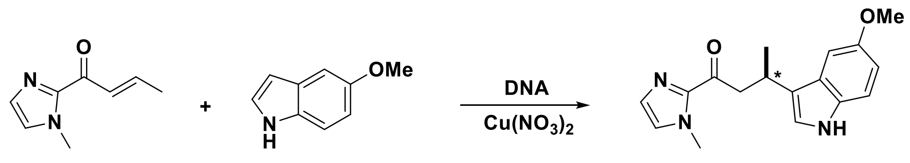

Table S1: Friedel-Crafts alkylation reaction using different catalytic conditions.[a]

Entry	DNA	DNA-structure	$Cu(NO_3)_2$ (mol%)	conv(%)[b]	*ee*(%)[b], [c]
1	-	-	-	<2	0
2	-	-	2.5	24	0
3	c-kit(wt)	GQ	-	<5	<-2
4	c-kit(wt)	GQ	2.5	34	<-5
5	c-kit(wt)	ds	-	<5	0
6	c-kit(wt)	ds	2.5	28	<+5
7	c-kit-T10	GQ	-	<5	<-2
8	c-kit-T10	GQ	2.5	45	<+5
9	c-kit-T10	ds	-	<5	0
10	c-kit-T10	ds	2.5	21	<+5

[a] See the standard Friedel-Crafts alkylation reaction procedure for detailed reaction condition. All experiments were performed in triplicate. [b] Both conversion and *ee* were calculated by using chiral HPLC; results are reproducible within ±5%. [c] (+) and (-) symbols refer to isomer with low and high retention time, respectively, from chiral HPLC column.

5.5 Reaction control-2

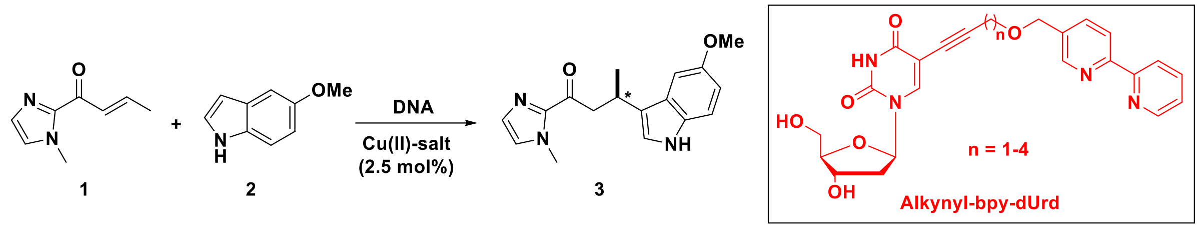

Table S2: Friedel-Crafts alkylation reaction using different catalytic conditions.[a]

Entry	DNA	DNA-structure	Modified nucleoside added	Cu(II)-salt (2.5 mol%)	conv(%)[b]	*ee*(%)[b], [c]
1	-	-	-	Cu-bpy	38	0
2	c-kit(wt)	GQ	-	Cu-bpy	43	+9
3	c-kit(wt)	ds	-	Cu-bpy	34	+15
4	c-kit-T10	GQ	-	Cu-bpy	52	+15
5	c-kit-T10	ds	-	Cu-bpy	41	+17
6	-	-	Propargyl-bpy-dUrd	$Cu(NO_3)_2$	29	0
7	c-kit(wt)	GQ	Propargyl-bpy-dUrd	$Cu(NO_3)_2$	32	+7
8	c-kit(wt)	ds	Propargyl-bpy-dUrd	$Cu(NO_3)_2$	15	<+5
9	c-kit-T10	GQ	Propargyl-bpy-dUrd	$Cu(NO_3)_2$	39	+16
10	c-kit-T10	ds	Propargyl-bpy-dUrd	$Cu(NO_3)_2$	22	+13
11	-	-	Butynyl-bpy-dUrd	$Cu(NO_3)_2$	31	0
12	c-kit(wt)	GQ	Butynyl-bpy-dUrd	$Cu(NO_3)_2$	58	0
13	c-kit(wt)	ds	Butynyl-bpy-dUrd	$Cu(NO_3)_2$	42	0
14	c-kit-T10	GQ	Butynyl-bpy-dUrd	$Cu(NO_3)_2$	68	+7
15	c-kit-T10	ds	Butynyl-bpy-dUrd	$Cu(NO_3)_2$	53	<+5
16	-	-	Pentynyl-bpy-dUrd	$Cu(NO_3)_2$	32	0
17	c-kit(wt)	GQ	Pentynyl-bpy-dUrd	$Cu(NO_3)_2$	43	<+5
18	c-kit(wt)	ds	Pentynyl-bpy-dUrd	$Cu(NO_3)_2$	34	<+5

19	c-kit-T10	GQ	Pentynyl-bpy-dUrd	$Cu(NO_3)_2$	56	+9
20	c-kit-T10	ds	Pentynyl-bpy-dUrd	$Cu(NO_3)_2$	38	+7
21	-	-	Hexynyl-bpy-dUrd	$Cu(NO_3)_2$	32	0
22	c-kit(wt)	GQ	Hexynyl-bpy-dUrd	$Cu(NO_3)_2$	51	0
23	c-kit(wt)	ds	Hexynyl-bpy-dUrd	$Cu(NO_3)_2$	31	0
24	c-kit-T10	GQ	Hexynyl-bpy-dUrd	$Cu(NO_3)_2$	64	<+5
25	c-kit-T10	ds	Hexynyl-bpy-dUrd	$Cu(NO_3)_2$	49	<+5

[a] See the standard Friedel-Crafts alkylation reaction procedure for detailed reaction condition. All experiments were performed in triplicate. [b] Both conversion and *ee* were calculated by using chiral HPLC; results are reproducible within ±5%. [c] (+) and (-) symbols refer to isomer with low and high retention time respectively from chiral HPLC column.

6 CD spectroscopy

An aqueous stock solution containing 5 nmol of the oligonucleotide was lyophilized first. Then the lyophilized DNA was dissolved in 500 µL of a solution of 3-(N-morpholino)propanesulfonic acid (MOPS) buffer (20 mM, pH 7) containing KCl (100 mM) to obtain the final DNA concentration 10 µM. The solution was equally divided into 2 parts. To one 250 µL DNA solution, 2.5 µL $Cu(NO_3)_2$ (1 mM) solution was added so that DNA:Cu(II) was 1:1. The other 250 µL DNA solution was used without addition of Cu(II). Both solutions were heated for 5 min at 90 °C, and slowly cooled down to room temperature. The solutions were kept at 5 °C overnight before collecting the CD spectra. The CD spectra were recorded on a Jasco model J-810 spectropolarimeter at 25 °C. The CD spectra were collected starting from 350 nm to 220 nm with 0.1 nm steps, 1 nm band width, 50 nm/min scanning speed and 0.1 cm optical path length. The data was averaged over 5 scans and a blank was subtracted.

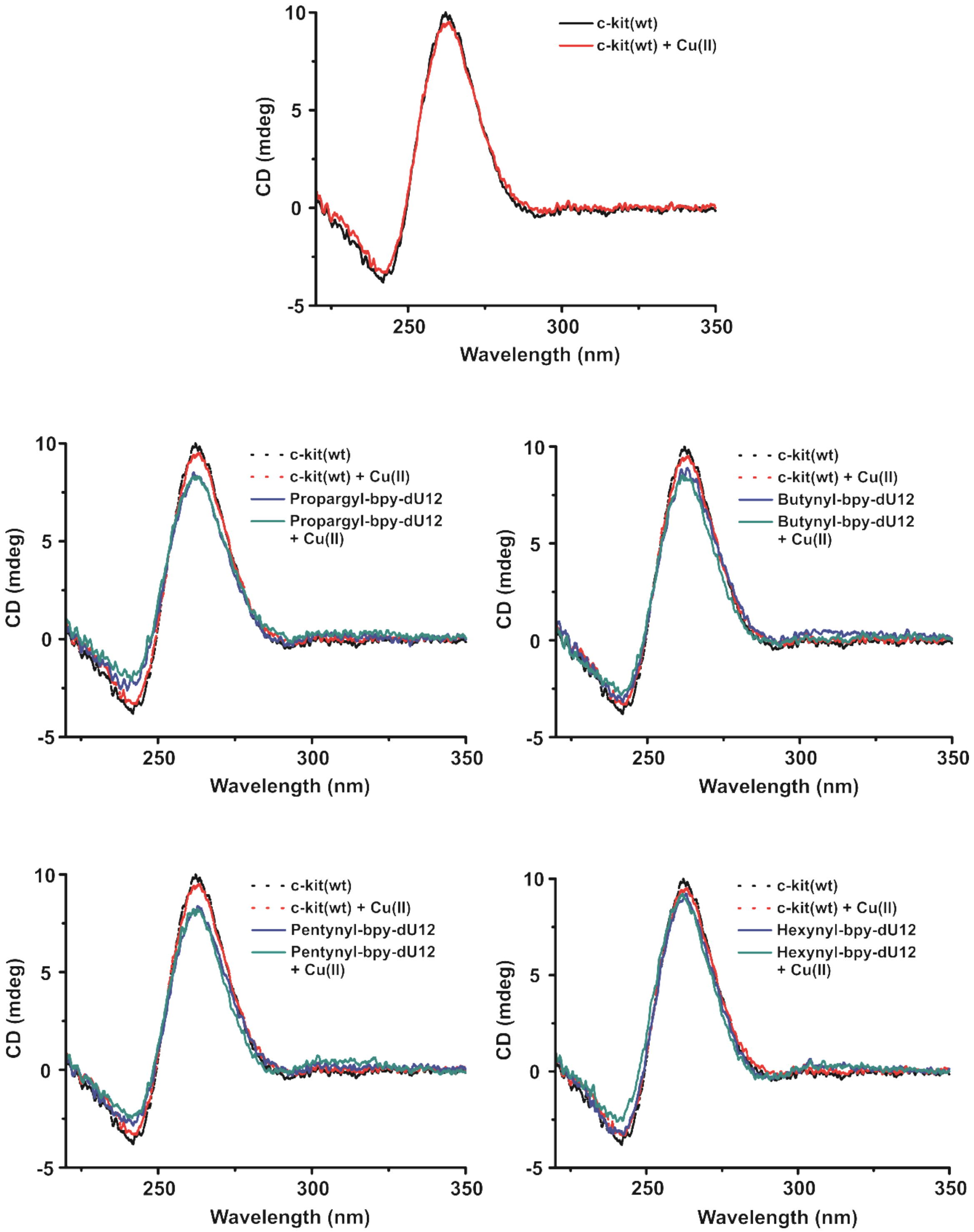

c-kit(wt)
c-kit(wt) + Cu(II)
CD (mdeg)
Wavelength (nm)
Propargyl-bpy-dU12
Propargyl-bpy-dU12 + Cu(II)
Butynyl-bpy-dU12
Butynyl-bpy-dU12 + Cu(II)
Pentynyl-bpy-dU12
Pentynyl-bpy-dU12 + Cu(II)
Hexynyl-bpy-dU12
Hexynyl-bpy-dU12 + Cu(II)

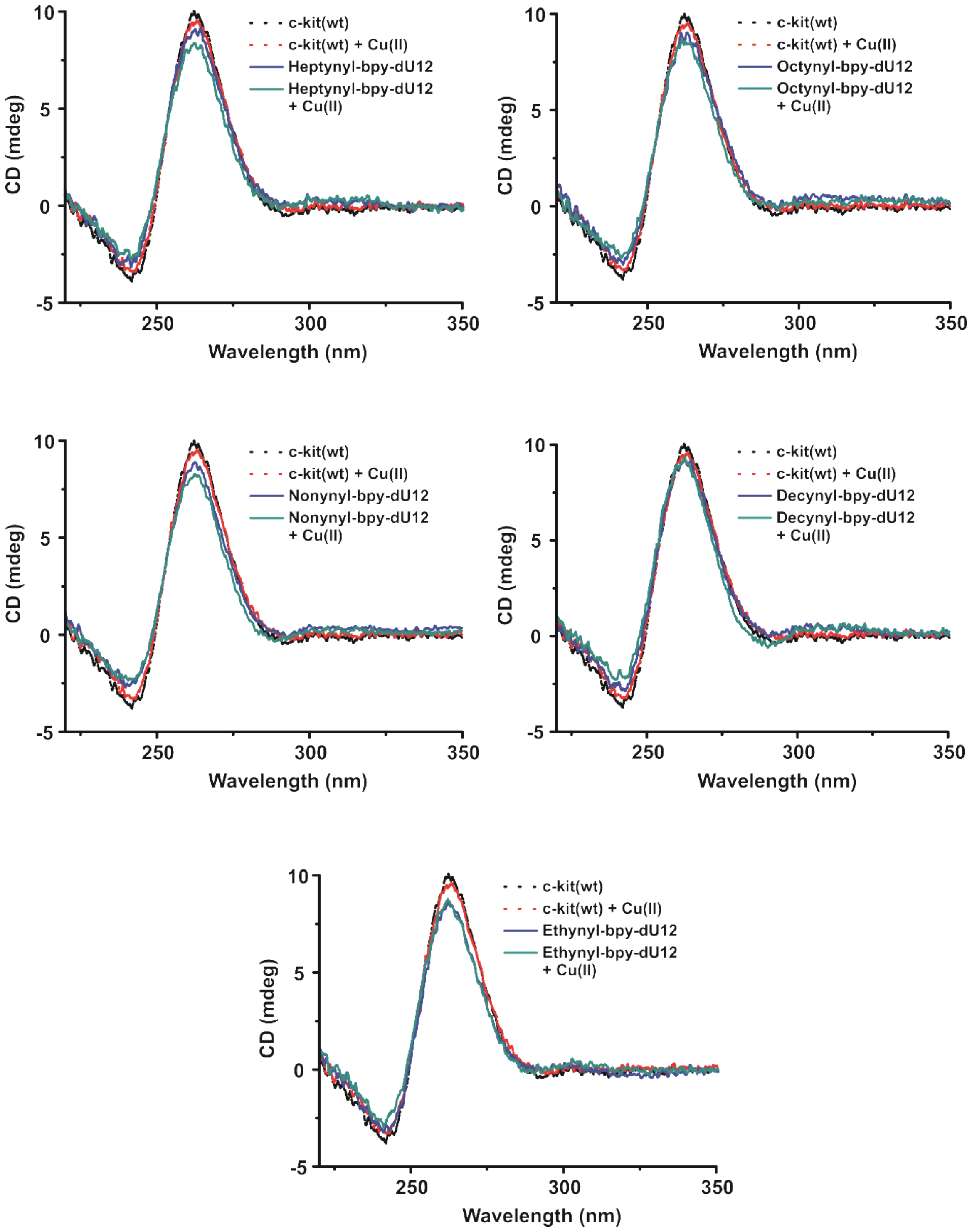

Figure S2: CD-spectra of c-kit(wt) oligonucleotide modified at position 12 in 20 mM MOPS (pH=7) + 100 mM KCl. All formed G-quadruplexes.[2,3]

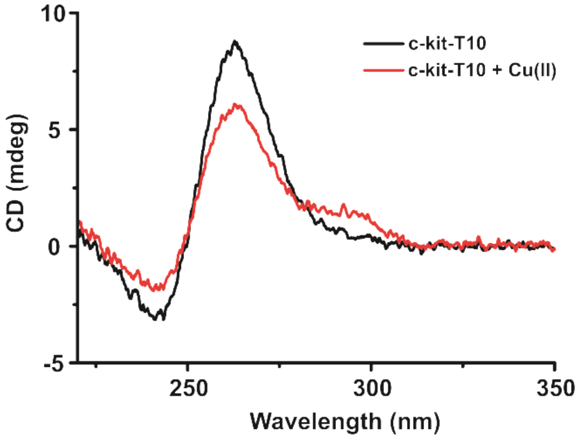
CD (mdeg)
Wavelength (nm)
c-kit-T10
c-kit-T10 + Cu(II)

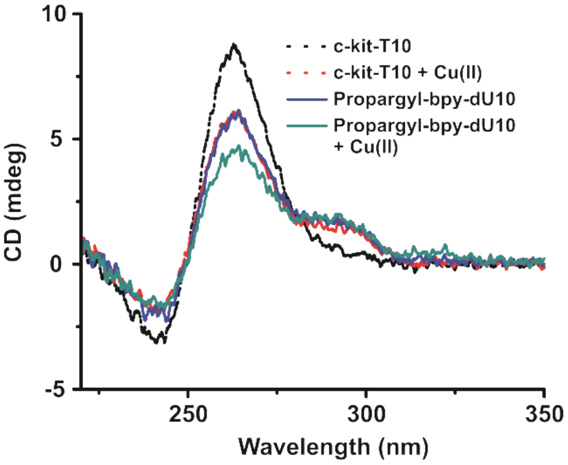
CD (mdeg)
Wavelength (nm)
c-kit-T10
c-kit-T10 + Cu(II)
Propargyl-bpy-dU10
Propargyl-bpy-dU10 + Cu(II)

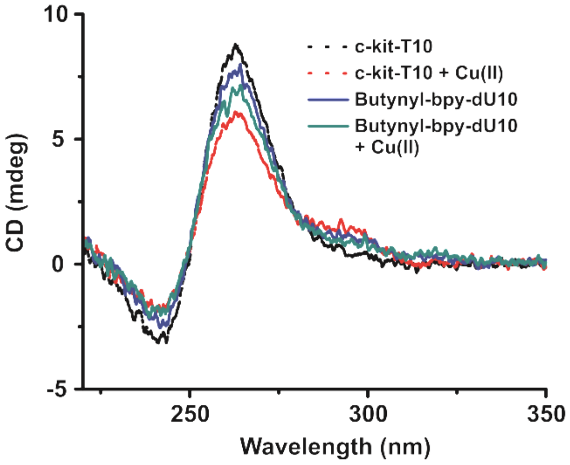
CD (mdeg)
Wavelength (nm)
c-kit-T10
c-kit-T10 + Cu(II)
Butynyl-bpy-dU10
Butynyl-bpy-dU10 + Cu(II)

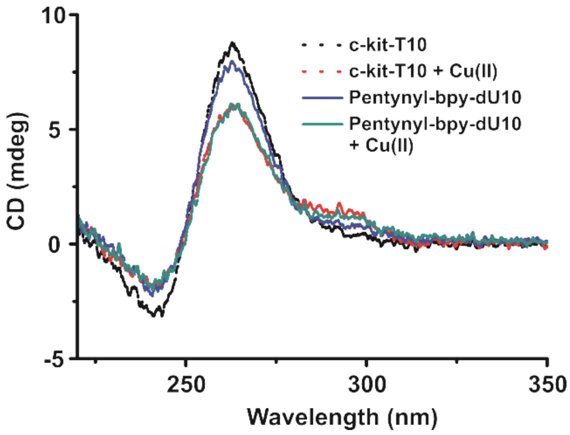
CD (mdeg)
Wavelength (nm)
c-kit-T10
c-kit-T10 + Cu(II)
Pentynyl-bpy-dU10
Pentynyl-bpy-dU10 + Cu(II)

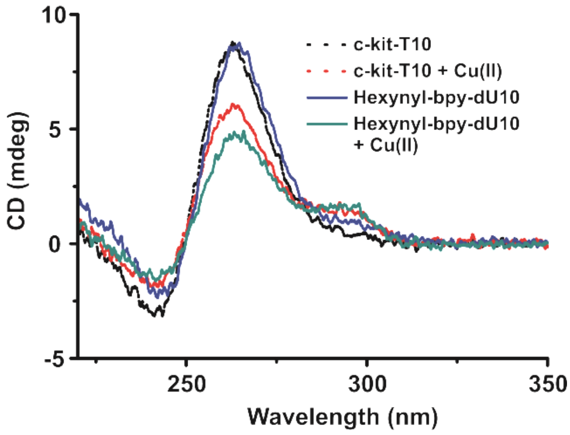
CD (mdeg)
Wavelength (nm)
c-kit-T10
c-kit-T10 + Cu(II)
Hexynyl-bpy-dU10
Hexynyl-bpy-dU10 + Cu(II)

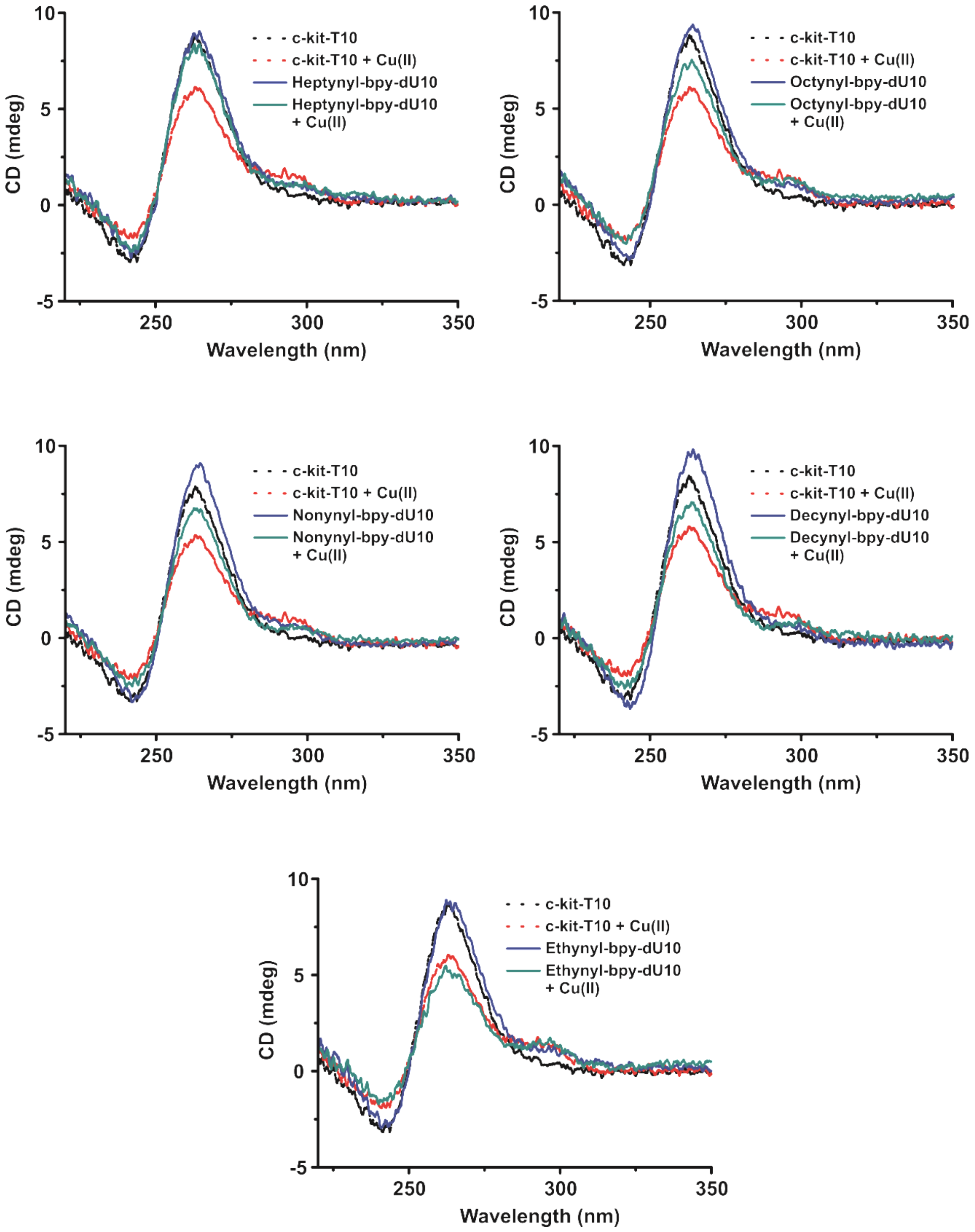

Figure S3: CD-spectra of c-kit oligonucleotide modified at position 10 in 20 mM MOPS (pH=7) + 100 mM KCl. All formed G-quadruplexes.[2,3]

7 Ethynyl-bpy modified DNA

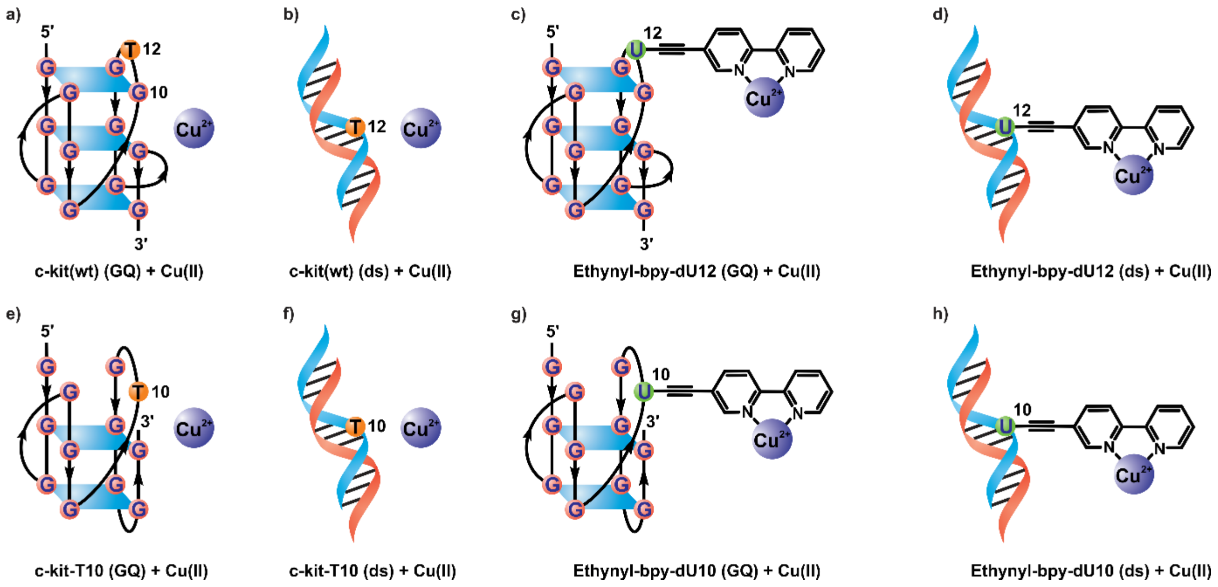

Figure S4. Folding of different G-quadruplex and dsDNA sequences in the presence of Cu(II).

a) Folding of (GQ) c-kit(wt) DNA as observed.[5,6] b) Schematic representation of hypothetical folding and metal binding of (ds) c-kit(wt). c) Schematic representation of hypothetical folding and metal binding of (GQ) ethynyl-bpy-dU12 DNA.[3] d) Schematic representation of hypothetical folding and metal binding of (ds) ethynyl-bpy-dU12 DNA. e) Schematic representation of hypothetical folding of (GQ) c-kit-T10 DNA. f) Schematic representation of hypothetical folding of (ds) c-kit-T10 DNA. g) Schematic representation of hypothetical folding and metal binding of (GQ) ethynyl-bpy-dU10 DNA.[3] h) Schematic representation of hypothetical folding and metal binding of (ds) ethynyl-bpy-dU10 DNA. All double-stranded (ds) samples included 1 equivalent of corresponding complementary strand.

8 References

1. Evans, D.A.; Fandrick, K.R.; Song, H.-J. Enantioselective Friedel−Crafts Alkylations of α,β-Unsaturated 2-Acyl Imidazoles Catalyzed by Bis(oxazolinyl)pyridine−Scandium(III) Triflate Complexes. *J. Am. Chem. Soc.* **2005**, *127*, 8942-8943.
2. Dey, S.; Jäschke, A. Tuning the Stereoselectivity of a DNA-Catalyzed Michael Addition through Covalent Modification. *Angew. Chem., Int. Ed.* **2015**, *54*, 11279-11282.
3. Dey, S.; Rühl, C.L.; Jäschke, A. Catalysis of Michael Additions by Covalently Modified G-Quadruplex DNA. *Chem.--Eur. J.* **2017**, *23*, 12162-12170.
4. Kalachova, L.; Pohl, R.; Hocek, M. Synthesis of 2′-Deoxyuridine and 2′-Deoxycytidine Nucleosides Bearing Bipyridine and Terpyridine Ligands at Position 5. *Synthesis* **2009**, 10.1055/s-0028-1083266, 105-112.
5. Phan, A.T.; Kuryavyi, V.; Burge, S.; Neidle, S.; Patel, D.J. Structure of an unprecedented G-quadruplex scaffold in the human c-kit promoter. *J. Am. Chem. Soc.* **2007**, *129*, 4386-4392.
6. Wei, D.G.; Parkinson, G.N.; Reszka, A.P.; Neidle, S. Crystal structure of a c-kit promoter quadruplex reveals the structural role of metal ions and water molecules in maintaining loop conformation. *Nucleic Acids Res.* **2012**, *40*, 4691-4700.
